# The transcription factor c-Fos coordinates with histone lysine-specific demethylase 2A to activate the expression of *cyclooxygenase-2*

**DOI:** 10.18632/oncotarget.5474

**Published:** 2015-09-25

**Authors:** Shaoli Lu, Yang Yang, Yipeng Du, Lin-lin Cao, Meiting Li, Changchun Shen, Tianyun Hou, Ying Zhao, Haiying Wang, Dajun Deng, Lina Wang, Qihua He, Wei-Guo Zhu

**Affiliations:** ^1^ Key Laboratory of Carcinogenesis and Translational Research (Ministry of Education), State Key Laboratory of Natural and Biomimetic Drugs, Beijing Key Laboratory of Protein Posttranslational Modifications and Cell Function, Department of Biochemistry and Molecular Biology, Peking University Health Science Center, Beijing 100191, China; ^2^ Department of Etiology, Peking University School of Oncology and Beijing Cancer Hospital & Institute, Beijing 100142, China; ^3^ Center of Medical and Health Analysis, Peking University Health Science Center, Beijing 100871, China; ^4^ Peking University-Tsinghua University Joint Center for Life Sciences, Beijing 100751, China

**Keywords:** COX-2, c-Fos, KDM2A, histone methylation

## Abstract

*Cyclooxygenase-2 (COX-2)* is overexpressed in a variety of human epithelial cancers, including lung cancer, and is highly associated with a poor prognosis and a low survival rate. Understanding how *COX-2* is regulated in response to carcinogens will offer insight into designing anti-cancer strategies and preventing cancer development. Here, we analyzed *COX-2* expression in several human lung cancer cell lines and found that *COX-2* expression was absent in the H719 and H460 cell lines by a DNA methylation-independent mechanism. The re-expression of *COX-2* was observed after 12-O-tetradecanoylphorbol-13-acetate (TPA) treatment in both cell lines. Further investigation found that H3K36 dimethylation was significantly reduced near the *COX-2* promoter because histone demethylase 2A (KDM2A) was recruited to the *COX-2* promoter after TPA treatment. In addition, the transcription factor c-Fos was found to be required to recruit KDM2A to the *COX-2* promoter for reactivation of *COX-2* in response to TPA treatment in both the H719 and H460 cell lines. Together, our data reveal a novel mechanism by which the carcinogen TPA activates *COX-2* expression by regulating H3K36 dimethylation near the *COX-2* promoter.

## INTRODUCTION

Lung cancer is the leading cause of cancer-related mortality in the world. The overall 5-year survival rate is approximately 15% for lung cancer patients, and this rate has remained largely unchanged for the last three decades [1, 2]. Even when this cancer is detected early, up to 50% of patients with pathologic stage I disease eventually relapse post-resection, and the median survival is less than one year [3]. Therefore, there is an urgent need to find a novel strategy that offers significant improvements to prolong the survival of lung cancer patients.

Cyclooxygenase-2 (COX-2), an isoform of cyclooxygenase, is the rate-limiting enzyme in the synthesis of prostaglandins. It is an immediate-early response gene not expressed in most normal tissues but can be induced by cytokines, growth factors, phorbol esters, oncogenes and chemical carcinogens [4–6]. For example, 12-O-tetradecanoylphorbol-13-acetate (TPA) was reported to up-regulate the expression of the *COX-2* gene in the human lung cancer cell line A549 [6]. *COX-2* plays a significant role in lung cancer carcinogenesis, promotion, angiogenesis, invasion and metastasis [7]; therefore, *COX-2* has become an important target being evaluated for lung cancer therapy and chemoprevention [8]. Understanding the *COX-2* expression mechanism is therefore important for developing strategies to prevent and treat lung cancer.

The transcription factors upstream of COX-2, such as AP-1, p300/CBP and NF-κB, can be activated by a series of kinase reactions after certain types of stimulation [9, 10]. The Fos family of transcription factors includes c-Fos, FosB, Fra-1 and Fra-2, as well as smaller FosB splice variants Fos B2 and delta FosB2 [11]. The Fos family genes encode leucine zipper proteins that can dimerize with Jun family proteins to form the transcription factor complex activating protein 1 (AP-1) [12]. c-Fos is a constituent of the first studied AP-1 protein complexes and is frequently overexpressed in tumor cells [11, 13]. c-Fos has also been reported to increase the expression of *COX-2* [4], but it is not clear how c-Fos activates *COX-2* expression.

Epigenetic control of cancer-related gene expression plays a critical role in cancer development [14]. In mammalian cells, epigenetic modifications of cytosine residues of the DNA CpG dinucleotide and ε-amino residues of histones have emerged as the major determinants of chromatin remodeling and gene transcriptional regulation [15–17]. Both DNA methylation and histone modifications coordinate to regulate gene expression. DNA methylation, which occurs in the CpG islands in the gene promoter region, is strongly related to gene inactivation [18, 19]. The absence of *COX-2* expression is closely correlated with the DNA methylation of the *COX-2* promoter, and 5-aza-2′-deoxycytidine, a DNA demethylating agent, can reactivate the expression of *COX-2* [20, 21]. In addition to DNA methylation, the modification of histones such as H3K4, H3K9 and H3K27 has also been reported to be an important factor in regulating the expression of *COX-2* [17, 22–25]. In contrast to other histone methylation sites, the effect of H3K36 methylation on *COX-2* gene expression has not been extensively studied. H3K36me2 has been reported to be enriched in the promoters of both expressed and silenced genes and thus plays an important role in the regulation of gene expression [17, 26, 27]. Several lysine methyltransferases (KMTs) and lysine demethylases (KDMs) are involved in H3K36 methylation [28]. For example, lysine-specific demethylase 2A (KDM2A) (also named JHDM1A) can demethylate H3K36me1/2 [29]. Whether there is a causal relationship between the enrichment of histone methylation-related enzymes such as KDM2A on the *COX-2* promoter and *COX-2* gene activation is worth investigating.

In this study, we analyzed the *COX-2* expression in several human lung cancer cell lines and found that the *COX-2* gene could be reactivated by TPA without affecting the DNA methylation status of the *COX-2* promoter. H3K36 dimethylation was significantly changed around the *COX-2* promoter after TPA treatment, and the changes in the H3K36 dimethylation were mediated by the transcription factor c-Fos, which recruited KDM2A to the *COX-2* promoter.

## RESULTS

### 12-O-tetradecanoylphorbol-13-acetate (TPA) activates the re-expression of the *COX-2* gene

**Figure 1:**
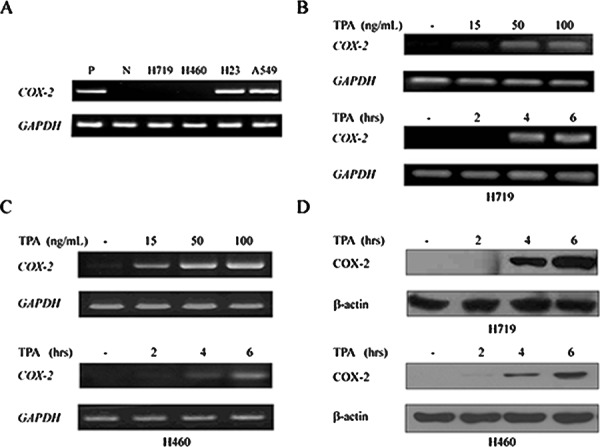
12-O-tetradecanoylphorbol-13-acetate (TPA) activates the re-expression of *COX-2* gene **A.** mRNAs from the human lung cancer cell lines H719, H460, H23 and A549 were extracted to detect *COX-2* expression by RT-PCR. GAPDH was used as a loading control. P indicates a positive control, and N indicates a negative control. **B.** H719 cells were treated with TPA for 6 hrs at different doses (0, 15, 50 and 100 ng/mL), or for different times (0, 2, 4 and 6 hrs) at 100 ng/mL. RT-PCR was performed to detect *COX-2* mRNA levels after treatment. GAPDH was used as a loading control. **C.** H460 cells were treated with TPA for 6 hrs at different doses (0, 15, 50 and 100 ng/mL), or for different times (0, 2, 4 and 6 hrs) at 100 ng/mL. RT-PCR was performed to detect the *COX-2* mRNA levels after treatment. GAPDH was used as a loading control. **D.** H719 and H460 cells were treated with TPA for different times (0, 2, 4 and 6 hrs) at 100 ng/mL, and proteins were extracted to detect the expression of COX-2 at the protein level by Western blotting. β-actin is shown as a loading control.

The *COX-2* gene is typically silent in most normal tissues and is overexpressed in many solid tumors, including lung cancer [7, 30]. To investigate the *COX-2* expression status in human lung cancer cell lines, RT-PCR was performed in H719, H460, H23 and A549 cells. As shown in Figure 1A, *COX-2* expression was absent in H719 and H460 cells but was high in H23 and A549 cells. To determine whether the *COX-2* gene could be re-expressed, 12-O-tetradecanoylphorbol-13-acetate (TPA), a potent carcinogen, was administered to both the H719 and H460 cell lines. *COX-2* expression was reactivated by TPA in a dose- and time-dependent manner in H719 and H460 cells as demonstrated by RT-PCR (Figure 1B and 1C). Reactivation of the *COX-2* gene peaked at 4–6 hrs after TPA treatment in both the H719 and H460 cells (Figure 1B and 1C), which was consistent with its protein levels as demonstrated by Western blotting (Figure 1D). These data indicate that *COX-2* can be reactivated by TPA in human lung cancer cells.

### TPA reactivates *COX-2* expression without affecting the DNA methylation status of its promoter but is associated with H3K36 methylation

**Figure 2:**
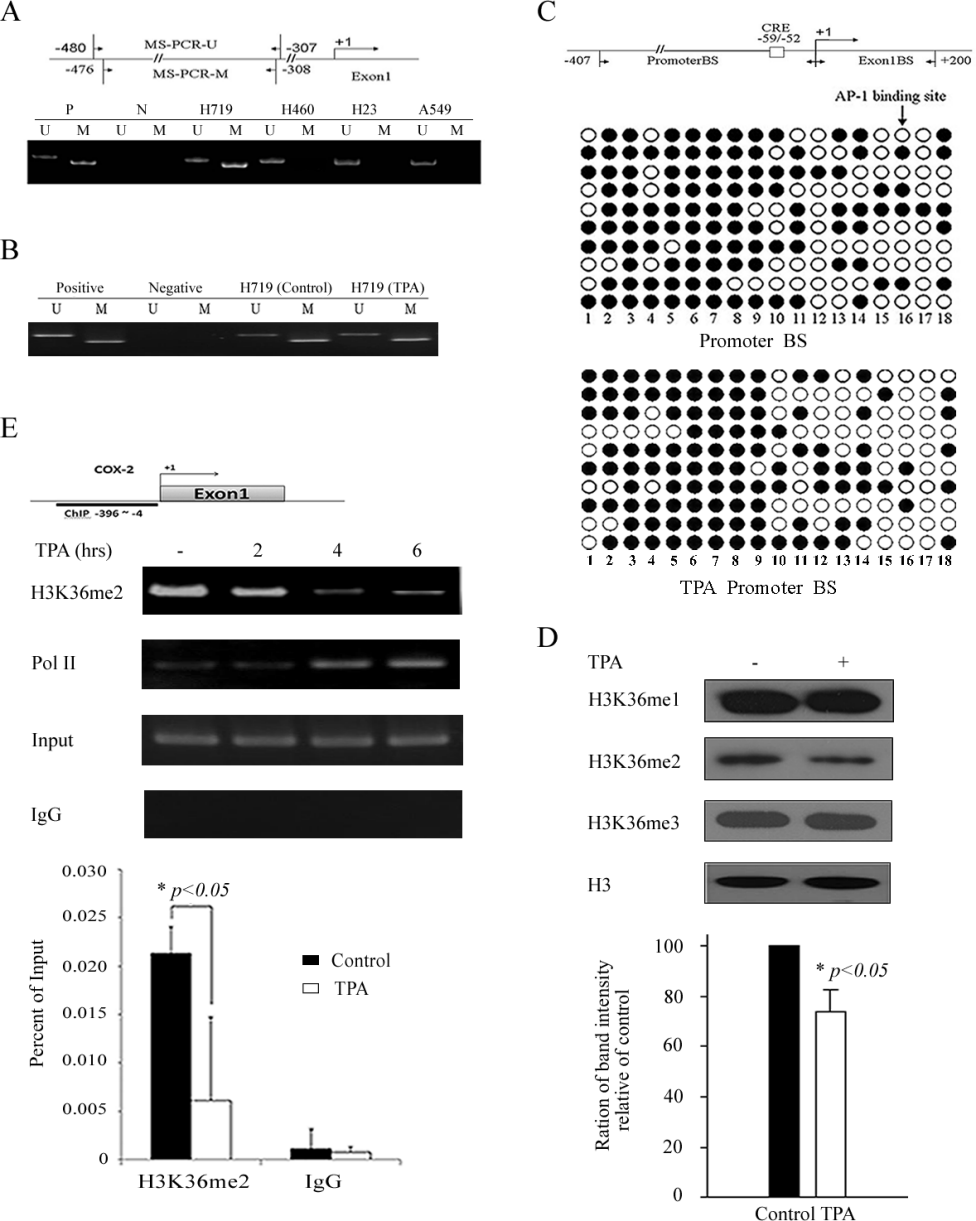
TPA reactivates *COX-2* expression without affecting the DNA methylation status of its promoter but associates with H3K36 methylation **A.** DNA methylation status of the *COX-2* promoter in H719, H460, H23 and A549 cells was detected by MSP. A schematic of the *COX-2* promoter and regions (−480 to −307, −476 to −308) selected for DNA methylation detection. M indicates a methylated band and U indicates an unmethylated band. **B.** H719 cells were treated with TPA for 6 hrs at 100 ng/mL, then MSP was used to detect the DNA methylation status of the *COX-2* promoter before and after TPA treatment. M indicates a methylated band and U indicates an unmethylated band. **C.** Bisulfite sequencing analysis of the *COX-2* promoter, which contains 18 CpG dinucleotides in H719 cells. A schematic of two regions, referred to as Promoter BS (−407 to +1) and Exon1 BS (+1 to +200), that were selected for bisulfate sequencing detection. AP-1 binding sites were labeled as CRE. More than ten independent clones of this cell line were sequenced. The promoter region showed 62.0% (112 methylated at a total of 180 sites) and 60.6% (109 methylated at a total of 180 sites) methylation before and after TPA treatment in H719 cells, respectively. Filled circle indicates methylated sites, and open circle indicates unmethylated sites. **D.** H719 cells were treated with 100 ng/mL TPA for 6 hrs, followed by Western blotting to determine the changes in H3K36 methylation patterns. H3 is shown as a loading control. H3K36me2 bands were scanned, and the relative band intensities were normalized for each H3 band. The band intensity of the control was set as 100; the numerical value of the intensity of each band represents the percentage of the band intensity with respect to that of the control. The representative value in the lower column diagram is an average relative band intensity of H3K36me2, with standard error shown from three independent experiments. *, *p* < 0.05. **E.** H719 cells were treated with TPA at 100 ng/mL for various intervals (0, 2, 4 and 6 hrs) and harvested for a ChIP assay to detect the enrichment of H3K36 me2 around the *COX-2* promoter (the upper panel). The bands containing anti-IgG served as negative controls. ChIP-qPCR assay was also used to detect the changes in the enrichment of H3K36 me2 at the *COX-2* promoter after 4 hrs of TPA treatment (the lower panel). *, *p* < 0.05.

Because the DNA hypermethylation of the *COX-2* promoter is related to *COX-2* gene silencing [21], we assessed the DNA methylation status of this promoter in the H719, H460, H23 and A549 cell lines. Genomic DNA from these human lung cancer lines was extracted and treated with sodium bisulfite for methylation-specific PCR (MSP) analysis. As shown in Figure 2A, the methylated band was clearly detected in H719 cells, which indicates a hypermethylated *COX-2* promoter in H719 cells. Conversely, unmethylated bands were detected in the H460, H23 and A549 cells (Figure 2A). Remarkably, the *COX-2* gene was absent in H460 cells, but the DNA methylation status of the *COX-2* promoter in this cell line was unmethylated, which suggests that DNA methylation might not be related to *COX-2* gene expression in H460 cells. To further clarify the effect of DNA methylation on *COX-2* gene expression, the alteration of DNA methylation status of the *COX-2* promoter was assessed before and after TPA treatment in H719 cells. H719 cells were treated with TPA (100 ng/mL) for 6 hrs, and the genomic DNA was extracted for MSP. Surprisingly, the DNA methylation status of the *COX-2* promoter was unchanged after TPA treatment as detected by MSP (Figure 2B). Consistently, this unaltered methylation pattern was also confirmed by bisulfite sequencing analysis. The methylated CpG levels within the *COX-2* promoter, which contains 18 CpG dinucleotides, were 62.0% and 60.6% before and after TPA treatment in H719 cells, respectively (Figure 2C). To clarify whether TPA treatment induced a change in the binding activity of methyl-CpG binding domain (MBD) proteins on the *COX-2* promoter, a chromatin immunoprecipitation (ChIP) assay was performed to detect the association between the MBD proteins and the *COX-2* promoter. As shown in Supplementary Figure S1, there were no obvious changes in the binding activity of the methyl-CpG binding domain protein 1 (MBD1) or the methyl-CpG binding domain protein 2 (MBD2) to the *COX-2* promoter before or after TPA treatment. This result was consistent with the data obtained from a quantitative real-time PCR for the ChIP assay (ChIP-qPCR assay) (Supplementary Figure S1). These results demonstrate that the reactivation of *COX-2* expression induced by TPA does not occur by altering the DNA methylation status of its promoter.

To investigate whether histone methylation is involved in the *COX-2* reactivation after TPA treatment, H719 cells were treated with 100 ng/mL of TPA for 6 hrs and harvested to detect the histone methylation levels by Western blotting. We did not find obvious changes in several prominent histone methylations, such as H3K4, H3K9, H3K27, H3K79 or H4K20, before or after TPA treatment (Supplementary Figure S2). Interestingly, we found that H3K36me2 was significantly decreased after TPA treatment in H719 cells (Figure 2D). To confirm the H3K36me2 changes upon TPA stimulation, the enrichment of the H3K36me2 around the *COX-2* promoter was evaluated using a ChIP assay (Figure 2E). As shown in Figure 2E, H3K36me2 enrichment around the *COX-2* promoter was obviously decreased 4 hrs after TPA treatment as demonstrated by ChIP and ChIP-qPCR assays. These results suggest that the changes in the H3K36me2 levels on the *COX-2* promoter may be associated with the transcriptional reactivation of the *COX-2* gene after TPA treatment.

### Lysine (K)-specific demethylase 2A (KDM2A) is involved in the TPA-induced reactivation of *COX-2*

**Figure 3:**
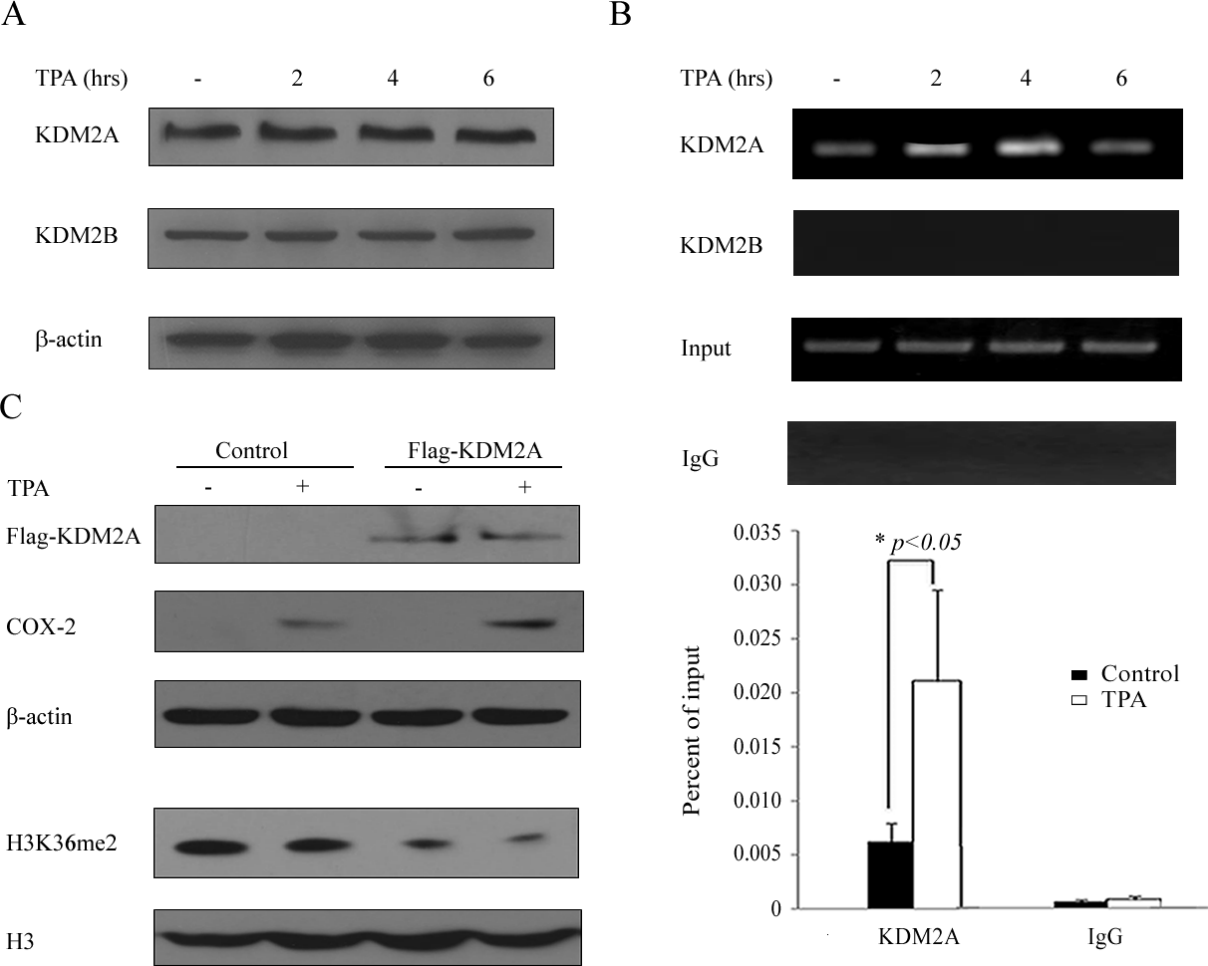
Lysine (K)-specific demethylase 2A (KDM2A) is involved in the TPA-induced reactivation of the *COX-2* gene **A.** H719 cells were treated with TPA at 100 ng/mL for various intervals (0, 2, 4 and 6 hrs), and then the cells were harvested for Western blotting to assess the expression of demethylases KDM2A and KDM2B. β-actin was used as a loading control. **B.** H719 cells were treated as outlined above and harvested for a ChIP assay to detect the binding activity of KDM2A and KDM2B around the *COX-2* promoter (the upper panel). The bands with anti-IgG served as negative controls. ChIP-qPCR assay was used to detect the binding activity changes of KDM2A around the *COX-2* promoter after 4 hrs of TPA treatment (the lower panel). *, *p* < 0.05. **C.** H719 cells were transfected with empty vector or with a Flag-KDM2A plasmid for 48 hrs and then treated with TPA for 6 hrs. The cells were harvested for Western blotting to detect the expression of *COX-2* and H3K36me2. β-actin and H3 were each used as loading controls.

We next searched for the enzymes that might be involved in the alteration of H3K36me2 after TPA treatment. H719 cells were treated with 100 ng/mL of TPA for up to 6 hrs and were harvested for Western blotting to detect the levels of H3K36me2 methyltransferases or demethylases. As shown in Figure 3A, the expression of the demethylases KDM2A (also called FBXL11) and KDM2B (also called NDY1) were not obviously changed after the TPA treatment. Additionally, the methyltransferases KMT3A (also called SETD2) and KMT3B (also called NSD1) and the demethylases KDM4A (also called JMJD2A) and KDM4B (also called JMJD2B) were also unchanged in TPA-treated cells (Supplementary Figure S3). Subsequently, a ChIP assay was used to detect the binding activity of histone methyltransferases or demethylases (for which an antibody was available) around the *COX-2* promoter upon TPA stimulation. As shown in Figure 3B, the KDM2A binding activity around the *COX-2* promoter was significantly increased in H719 cells and reached a peak at 4 hrs after TPA treatment. These results indicate that KDM2A may play an important role in the TPA-induced reactivation of *COX-2* by down-regulating H3K36me2 around the *COX-2* promoter. To further confirm the effect of KDM2A on *COX-2* reactivation, an empty vector or a Flag-KDM2A was transfected into H719 cells for 48 hrs, followed by treatment with TPA for 6 hrs to detect the H3K36me2 changes by Western blotting. As shown in Figure 3C, H3K36me2 was obviously decreased in the Flag-KDM2A-transfected H719 cells, and this decrease was more noticeable after TPA treatment. Moreover, the reactivation of *COX-2* was also more apparent in the Flag-KDM2A-transfected H719 cells after TPA treatment. These data indicate the important role of KDM2A in the TPA-induced decrease of the H3K36me2 and the reactivation of *COX-2* expression.

### c-Fos is required for the H3K36me2 reduction around the *COX-2* promoter

**Figure 4:**
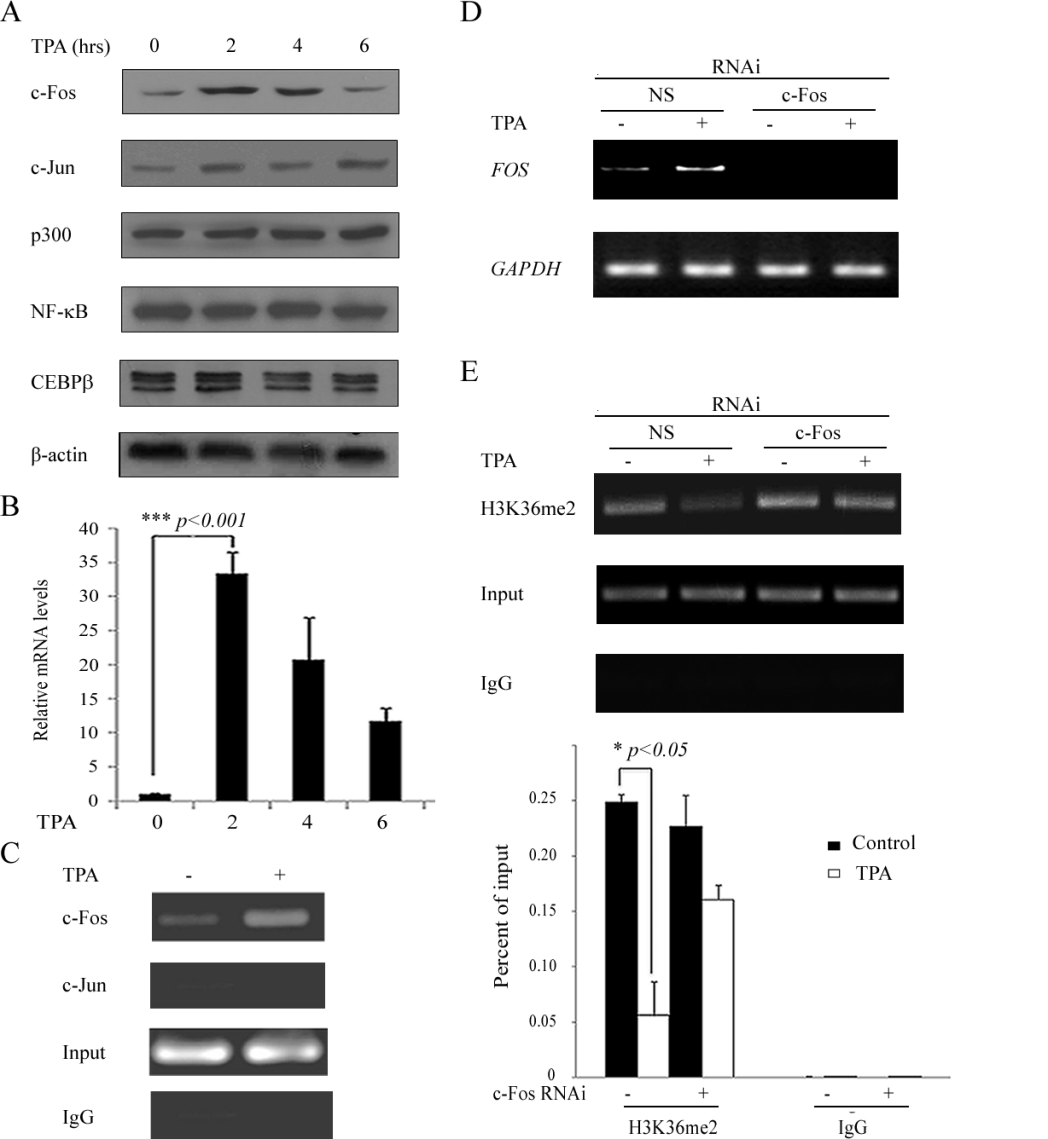
The upregulation of c-Fos is responsible for H3K36me2 reduction around the *COX-2* promoter by TPA treatment **A.** H719 cells were treated with TPA at 100 ng/mL for various intervals (0, 2, 4 and 6 hrs), and the cells were then harvested for Western blotting to detect the expression of c-Fos, c-Jun, p300, NF-κB and CEBPβ. β-actin was used as a loading control. **B.** H719 cells were treated as outlined above, and the expression of *FOS* on mRNA levels was confirmed by real-time PCR. ***, *p* < 0.001. **C.** H719 cells were treated with TPA at 100 ng/mL for 2 hrs and then harvested for a ChIP assay to detect the binding activity of c-Fos or c-Jun near the *COX-2* promoter. The bands with anti-IgG served as negative controls. **D.** H719 cells were transfected with c-Fos siRNA and non-specific siRNA (NS) for 24 hrs and then treated with TPA for 2 hrs at 100 ng/mL. RT-PCR was performed to detect *FOS* mRNA expression. GAPDH was used as a loading control. **E.** H719 cells were treated with TPA at 100 ng/mL for 4 hrs and harvested for a ChIP assay to detect the enrichment of H3K36me2 around the *COX-2* promoter (the upper panel). The bands with anti-IgG served as negative controls. ChIP-qPCR assay was used to detect the change of H3K36me2 at the *COX-2* promoter after 4 hrs of TPA treatment (the lower panel). *, *p* < 0.05.

The main upstream transcriptional factors of *COX-2*, such as AP-1, p300/CBP and NF-κB, can be activated by a series of kinase reactions after certain types of stimulation and can thus induce the expression of *COX-2* [9, 10]. To explore the effect of the related transcription factors on *COX-2* re-expression during TPA treatment, H719 cells were treated with TPA at 100 ng/mL for up to 6 hrs and then harvested to detect the expression of these transcriptional factors with Western blotting. As shown in Figure 4A, the protein levels of p300, NF-κB and CEBPβ were not changed after TPA treatment, but the expression of c-Fos and c-Jun, both main dimer units of AP-1, increased significantly in H719 cells. The c-Fos protein levels were increased significantly and reached a peak at 2 hrs after TPA treatment (Figure 4A). In addition, a qPCR was performed to detect *FOS* mRNA levels in H719 cells. The *FOS* mRNA levels also increased significantly and reached a peak at 2 hrs after TPA treatment in H719 cells (Figure 4B). To further clarify the effect of c-Fos on the expression of *COX-2*, a ChIP assay was performed to detect c-Fos binding activity around the *COX-2* promoter. As shown in Figure 4C, c-Fos binding activity around the *COX-2* promoter increased dramatically after TPA treatment in H719 cells. In addition, the binding activity of c-Jun around the *COX-2* promoter failed to be detected, which indicates that c-Jun may not be involved in the *COX-2* reactivation in H719 cells (Figure 4C). To confirm whether c-Fos is required for the reactivation of *COX*-2, H719 cells were transiently transfected with a siRNA against *FOS* or a non-specific siRNA and then treated with TPA. Cells were harvested and subjected to RT-PCR or a ChIP assay. As shown in Figure 4D, the expression of *FOS* induced by TPA significantly decreased in cells transfected with c-Fos siRNA compared with that in the cells transfected with the non-specific siRNA. In addition, the decreased H3K36me2 around the *COX-2* promoter was almost completely reversed after TPA treatment in the H719 cells with *FOS* expression knockdowned by c-Fos siRNA (Figure 4E). Additionally, the enriched H3K36me2 at the *COX-2* promoter was decreased by 75.0% in the non-specific siRNA-transfected H719 cells, whereas the changes in H3K36me2 at the *COX-2* promoter were largely reversed when *FOS* was knocked down by c-Fos siRNA, as demonstrated by ChIP-qPCR assay (Figure 4E). These data indicate that c-Fos is indispensable for the histone H3K36me2 down-regulation at the *COX-2* promoter and for the reactivation of the expression of *COX-2* after TPA treatment.

### c-Fos interacts with KDM2A *in vitro* and *in vivo*

**Figure 5:**
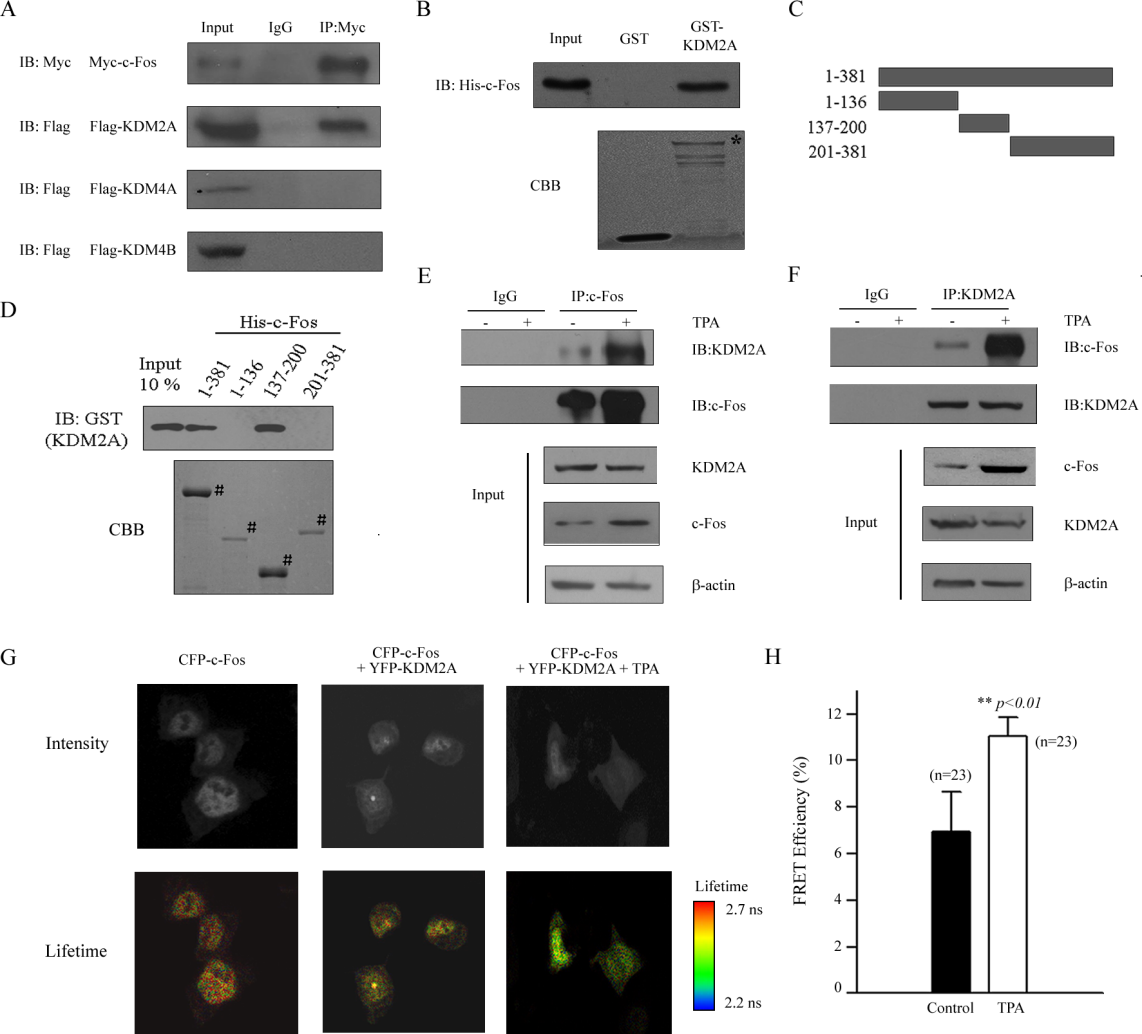
c-Fos interacts with KDM2A *in vitro* and *in vivo* **A.** A Myc-tagged c-Fos and a Flag-tagged histone modification enzyme (as indicated in the figure) were co-transfected into HEK293T cells and the proteins were then extracted for Co-IP with anti-Myc and anti-Flag antibodies, followed by immunoblotting with anti-Myc. **B.** GST-KDM2A was incubated with His-c-Fos, and Western blotting or Coomassie staining was performed to detect the direct binding of c-Fos and KDM2A *in vitro*. * indicates the specific bands. **C.** Schematic of plasmids encoding full-length c-Fos, an N-terminal fragment (aa 1–136), a middle fragment (aa 137–200) and a C-terminal fragment (aa 201–381). **D.** His-c-Fos FL or fragments were incubated with GST-KDM2A, and Western blotting with an anti-GST antibody or Coomassie staining was performed to detect the interaction. # indicates the specific bands. **E.** H719 cells were treated without or with TPA for 2 hrs at 100 ng/mL, then the cell were extracted for Co-IP using an anti-c-Fos antibody, followed by Western blotting using anti-KDM2A or anti-c-Fos antibodies to detect the interaction between c-Fos and KDM2A. **F.** H719 cells were treated without or with TPA for 2 hrs at 100 ng/mL, then the cells were extracted for Co-IP using anti-KDM2A antibody, followed by Western blotting using an anti-KDM2A or anti-c-Fos antibodies to detect the interaction between c-Fos and KDM2A. **G.** HEK293T cells were transfected with pECFP-c-Fos and/or pEYFP-KDM2A plasmids, followed by TPA treatment for 2 hrs. FLIM assays were performed, and the images of representative cells are shown for each group. **H.** FRET efficiency is shown in the column diagram, and the representative value is an average FRET efficiency of 23 cells with standard error. **, *p* < 0.01.

To explore the mechanism by which c-Fos regulates H3K36me2 reduction and its role in the reactivation of *COX-2* after TPA treatment in lung cancer cells, several demethylases that are involved in histone H3K36me2 demethylation were investigated by Co-IP (Figure 5A). A Myc-tagged c-Fos and Flag-tagged demethylases, including KDM2A, KDM4A and KDM4B, were co-transfected into HEK293T cells, and the interaction between c-Fos and each of these H3K36 demethylases was assessed. As shown in Figure 5A, KDM2A interacted with c-Fos, which implies that KDM2A may be the co-activator of c-Fos in the mediation of *COX-2* reactivation after TPA treatment. To confirm this result, a His-tagged c-Fos protein was expressed in bacteria, purified and incubated with GST and GST-KDM2A to investigate whether KDM2A directly interacted with c-Fos. As shown in Figure 5B, c-Fos showed direct interactions with GST-KDM2A but not with GST alone. To further map the regions of c-Fos responsible for its interaction with KDM2A, fragments representing full length (FL), N-terminal (aa 1–136), middle (aa 137–200) and C-terminal (aa 201–381) versions of c-Fos as shown in Figure 5C were expressed, purified and incubated with a GST-KDM2A. As shown in Figure 5D, GST-KDM2A specifically interacted with the FL and middle region fragment of c-Fos, showing that KDM2A can directly interact with c-Fos *in vitro*. Next, we explored the effect of the interaction between KDM2A and c-Fos after TPA stimulation. H719 cells were treated with or without TPA for 2 hrs, and the cells were then harvested to detect the endogenous interaction between KDM2A and c-Fos by Co-IP. As shown in Figure 5E and 5F, the interaction of KDM2A and c-Fos significantly increased after TPA treatment in H719 cells compared with the non-TPA treated control. Also, the similar result was found in H460 cells (Supplementary Figure S4F). In addition, a fluorescence resonance energy transfer (FRET) assay by fluorescence lifetime imaging (FLIM) was performed to confirm the interaction of KDM2A and c-Fos in response to TPA treatment. HEK293T cells were transfected with pECFP-c-Fos and/or pEYFP-KDM2A plasmids, followed by TPA treatment for 2 hrs. The donor lifetime (CFP-c-Fos) was shortened in the TPA treatment group compared with the control group (Figure 5G). Statistically, the energy transfer efficiency was obviously increased after TPA treatment in HEK293T cells compared with that in untreated cells, demonstrating that the interaction between KDM2A and c-Fos was increased in response to TPA treatment (Figure 5H). Taken together, this suggests that KDM2A directly interacts with c-Fos and that the increased interaction between KDM2A and c-Fos after TPA treatment plays a critical role in the reactivation of the *COX-2* gene expression.

### c-Fos recruits KDM2A to the *COX-2* promoter

**Figure 6:**
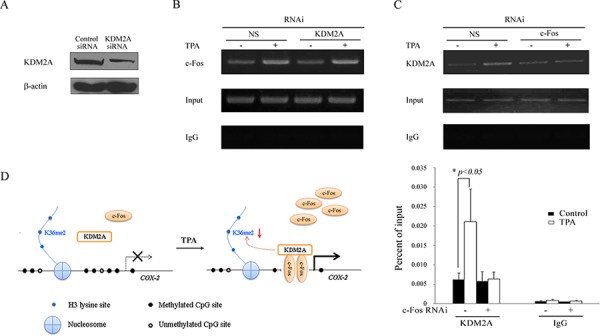
c-Fos recruits KDM2A to the *COX-2* promoter **A.** H719 cells were transfected with KDM2A siRNA or non-specific siRNA (NS) for 48 hrs, followed by Western blotting to assess the efficiency of the RNAi. **B.** H719 cells were transfected with KDM2A siRNA or non-specific siRNA (NS) for 48 hrs and treated with TPA at 100 ng/mL for 2 hrs. A ChIP assay was performed to detect the binding activity of c-Fos around the *COX-2* promoter. The bands containing anti-IgG served as negative controls. **C.** H719 cells were transfected with c-Fos siRNA or non-specific siRNA (NS) for 48 hrs and then treated with TPA for 4 hrs at 100 ng/mL. A ChIP assay was performed to detect the binding activity of KDM2A around the *COX-2* promoter (the upper panel). ChIP-qPCR assay was used to detect the change in binding activity of KDM2A around the *COX-2* promoter after 4 hrs of TPA treatment (the lower panel). *, *p* < 0.05. **D.** A schematic showing a possible mechanism by which TPA induces the methylated *COX-2* gene re-expression. All symbols are defined in the diagram.

To explore the effect of the interaction between KDM2A and c-Fos in cancer cells after TPA treatment, KDM2A expression was down-regulated by KDM2A siRNA (Figure 6A) to test the binding activity of c-Fos to the *COX-2* promoter. H719 cells were treated with TPA after transient transfection with KDM2A siRNA or a non-specific siRNA for 48 hrs, and the cells were then harvested and subjected to a ChIP assay. As shown in Figure 6B, c-Fos was enriched on the *COX-2* promoter by TPA treatment, which was not dependent on the presence of KDM2A. By contrast, the enrichment of KDM2A around the *COX-2* promoter was significantly reduced when *FOS* was knockdowned. As shown in Figure 6C, the enrichment of KDM2A around the *COX-2* promoter in non-specific siRNA-transfected cells was significantly increased after TPA treatment compared with that in the c-Fos siRNA-transfected H719 cells. The enrichment of KDM2A around the *COX-2* promoter was reduced by 71.0% after TPA treatment in c-Fos siRNA-transfected cells compared with that in the non-specific siRNA-transfected H719 cells, as assayed by ChIP-qPCR (Figure 6C). These results indicate that c-Fos reactivates *COX-2* expression by recruiting KDM2A to the *COX-2* promoter, which in turn decreases H3K36me2 around the *COX-2* promoter.

## DISCUSSION

In this study, our data reveal a novel mechanism by which the carcinogen TPA activates the expression of *COX-2* in human lung cancer cells. Histone demethylation at H3K36me2, but not DNA demethylation around the promoter of *COX-2*, is critical for the TPA-induced reactivation of *COX-2*. In addition, the transcription factor c-Fos plays an important role in the TPA-mediated reduction of histone H3K36 dimethylation by recruiting the H3K36 demethylase KDM2A onto the *COX-2* promoter.

Epigenetic mechanisms are involved in the response to a variety of environmental carcinogen exposures, yet the exact mechanisms underlying this effect are not well understood. Aberrant DNA methylation of the *COX-2* CpG islands was first reported in colorectal tumors [31], and DNA hypermethylation of *COX-2* at its promoter was shown to be related to *COX-2* gene silencing [20, 21]. However, in this study, DNA methylation was not changed before or after TPA treatment, showing that TPA-induced *COX-2* gene re-expression is independent of DNA methylation (Figure 2B). This finding is not so surprising, considering that TPA is not a demethylation agent, such as 5-aza-2′-deoxycytidine. When H719 cells were treated with 5-aza-2′-deoxycytidine, *COX-2* expression was reactivated in a DNA methylation-dependent manner (Supplementary Figure S5A and S5B). Also, the enrichment of H3K36me2 around the *COX-2* promoter was obviously decreased after 5-aza-2′-deoxycytidine treatment as detected by ChIP-qPCR assay, suggesting that H3K36me2 demethylation around the *COX-2* promoter plays some role in *COX-2* re-expression in H719 cells (Supplementary Figure S5C). However, whether the re-expression of *COX-2* after 5-aza-2′-deoxycytidine treatment is exclusively due to histone demethylation or DNA demethylation needs further exploration. The re-expression of *COX-2* induced by TPA or 5-aza-2′-deoxycytidine may occur through different mechanisms, and TPA reactivates *COX-2* mainly by a c-Fos- and KDM2A-mediated decrease of H3K36me2.

In addition to DNA methylation, histone modification is a critical epigenetic regulator that helps control chromatin structure and gene regulation [32]. Among histone modifications, histone methylation-mediated gene regulation has been well studied [32, 33]. The histone methylation of H3K4 and H3K79 plays a role in activating gene expression, whereas the methylation of H3K9 and H3K27 has been suggested to be a gene repressor in euchromatin [15, 32]. H3K36 methylation was reported to play dual functions in gene expression; studies in multiple systems have supported a role for H3K36 methylation in transcriptional activation [34, 35]. The levels and spatial distribution of di- and tri-H3K36 methylation in several genes have been analyzed, which showed that the active genes contain high levels of di- and tri- H3K36 methylation in actively transcribed regions [34]. Human epigenetic analysis also showed that the gene bodies of actively transcribed genes are associated with H3K36me3 [35]. By contrast, the effect of H3K36 methylation on transcriptional repression has also been reported [27, 36–38]. Set2 is a histone methyltransferase (HMT) that is site-specific for lysine 36 (Lys36) in the H3 tail. Set2 represses the basal expression of *GAL4* in *Saccharomyces cerevisiae* by mediating the methylation form of H3K36 [36]. H3K36me2 has been reported to recruit histone demethylase complex Rpd3s, which is one of the strong gene repression factors that represses spurious transcription [37]. In addition, the epigenetic inactivation of the histone methyltransferase NSD1 leads to the specifically diminished methylation of the histone lysine residues H3K36 and increases the expression of oncogene *MEIS1* in human neuroblastoma and glioma [38]. In our study, we found that H3K36me2 was reduced both in the promoter and the transcribed regions of the *COX-2* gene, indicating that H3K36me2 might play an important role in gene repression by mediating the transcriptional reactivation of *COX-2* in human lung cancer cells (Figure 2D and 2E, Supplementary Figure S4A and S4B, and Supplementary Figure S6A and S6B). In addition, the changes of H3K36me3 in the transcribed region of the *COX-2* gene were detected by ChIP-qPCR assay, and no significant increased H3K36me3 enrichment was found in each exon of the *COX-2* gene, suggesting that H3K36me3 is not related to the increased expression of *COX-2* in response to TPA treatment (Supplementary Figure S6C). Thus, H3K36 methylation appears to act as both an activating and an inhibitory signal, and the overall biological readout may depend on the context of the additional surrounding markers and their corresponding reader proteins.

H3K36 is methylated by lysine methyltransferases and demethylated by lysine demethylases, including KDM2A [28, 39]. KDM2A is a JmjC-containing histone demethylase that targets mono- and dimethylated Lys36 on H3 residues. In lung cancer, KDM2A was reported to be a novel oncogenic promoter of non-small cell lung cancer (NSCLC), and the overexpression of KDM2A was shown to promote tumorigenicity and metastasis by epigenetically enhancing ERK1/2 signaling in NSCLC [40]. KDM2A expression was found to be increased in gastric cancer tissues, and KDM2A may regulate the growth and motility of gastric cancer cells by down-regulating the expression of tumor suppressor programmed cell death 4 (PDCD4) in the progression of gastric cancer [41]. Conversely, KDM2A was found to regulate E2F1-mediated gene transcription and thus to suppress the invasion and migration in breast cancer cells [42]. Silencing KDM2A using small interfering RNAs increased the invasion and migration of breast cancer cells by suppressing a subset of matrix metalloproteinases, such as MMP-2, MMP-9, MMP-4 and MMP-15 [42]. In our study, the enrichment of KDM2A around the *COX-2* promoter was significantly increased after TPA treatment, which indicates that KDM2A is involved in the TPA-induced reactivation of the *COX-2* gene (Figure 3B and 3C). KDM2A may reactivate the expression of *COX-2* gene by decreasing H3K36me2 near the *COX-2* promoter in human lung cancer cells.

Chromatin-modifying enzymes function as co-factors and are frequently recruited by transcription factors to chromatin to activate or repress gene expression. Our data show that c-Fos was indispensable for histone H3K36me2 changes at the *COX-2* promoter by recruiting KDM2A to the *COX-2* promoter and reactivating *COX-2* expression after TPA treatment in both H719 and H460 cells (Figure 4–6 and Supplementary Figure S4C–S4G). The effects of c-Fos on histone modification have also been found in the regulation of other genes [43, 44]. c-Fos induced SIRT6 transcription, which repressed survivin by reducing histone H3K9 acetylation and NF-κB activation [43]. Oxidized low-density lipoprotein (oxLDL), which is a major risk factor for cardiovascular disease, could effectively increase the expression of microRNA-29b through the activation of c-Fos expression. Decreased methylation at H3K9 in response to oxLDL was significantly abolished in the absence of c-Fos, which implies that oxLDL-mediated histone modifications are mediated through the activation of c-Fos, which eventually causes miR-29b up-regulation [44]. These results are similar to our data showing that c-Fos is an important transcription factor for gene regulation through mediating histone modification. Also, several co-downstreams of both *COX-2* and c-Fos were identified after TPA treatment in H460 cells. The expression of *mPGES-1* and *EP2* is significantly increased in a dose-dependent manner after TPA treatment, which is similar with the pattern of *COX-2* expression under the same conditions. These data suggest that the COX-2—mPGES-1—EP2 pathway was activated in response to TPA treatment (Supplementary Figure S7A). Among the downstreams of c-Fos, we found that the expression pattern of VEGF was same as the expression pattern of c-FOS, while an opposite expression pattern was found in the expression of p27 after TPA stimulation (Supplementary Figure S7B). Interestingly, the expression of *MMP1* was constantly increased in a dose-dependent manner after TPA treatment, suggesting that *MMP1* may be another gene activated by TPA through c-Fos.

To our knowledge, this is the first report showing that transcription factor c-Fos mediates the TPA-induced *COX-2* reactivation by recruiting KDM2A to the *COX-2* promoter in human lung cancer cells. Based on the above observations, we propose a mechanism to explain the effect of TPA on *COX-2* gene reactivation (Figure 6D). TPA directly induces the expression of c-Fos and thus enriches c-Fos around the *COX-2* promoter. Subsequently, c-Fos recruits KDM2A to the *COX-2* promoter, which further decreases the H3K36me2 levels on the *COX-2* promoter. Consequently, *COX-2* is reactivated by TPA. Our data suggest a novel mechanism by which TPA activates the expression of *COX-2* and provide a new target for designing anti-cancer strategies and preventing lung cancer development.

## MATERIALS AND METHODS

### Cell culture and chemical treatment

Human lung cancer cell lines H719, H460, H23 and A549 were purchased from American Type Culture Collection (ATCC, Manassas, VA). H460 and H23 cells were cultured in RPMI-1640 medium supplemented with 10% heat-inactivated fetal bovine serum (FBS). H719 cells were cultured in DMEM: F12 medium with 5% heat-inactivated FBS. A549 cells were cultured in F-12K medium with 10% heat-inactivated FBS. All the cells were cultured in the medium mentioned above with penicillin/streptomycin in a 37°C incubator with a humidified, 5% CO2 atmosphere. All cells obtained from ATCC were immediately expanded and frozen down for future use (every 3 months from a frozen vial) of the same batch of cells. Chemical reagents 12-O-tetradecanoylphorbol-13-acetate (TPA) was purchased from Sigma (St Louis, MO, USA). TPA (dissolved in DMSO) was fresh added in medium and then incubated at 37°C for various time intervals. Control cells were treated with dimethyl sulfoxide for similar time intervals. The final concentration of added DMSO was lower than 0.1% (V/V).

### Methylation-specific PCR

DNA was extracted and then treated with bisulfite as previously described with minor modifications [45]. Briefly, genomic DNA (1 μg) in a volume of 50 μl was denatured with NaOH (final concentration, 0.275 M) for 10 min at 42°C. The denatured DNA was then treated with 10 μl of 10 mM hydroquinone and 520 μl of 3 M sodium bisulphate at 50°C overnight. The MSP primers for *COX-2* were as follows: Methylated forward primer, 5′-CGT ATA GAT TAG ATA CGG C-3′ and methylated reverse primer, 5′-TTT ACC CGA ACG CTT CCG AA-3′; Unmethylated forward primer, 5′-AAG ATG TAT AGA TTA GAT ATG GT-3′and unmethylated reverse primer, 5′-CTT TAC CCA AAC ACT TCC AAA-3′. The PCR products were then size fractionated by using a 2% agarose gel, stained with ethidium bromide, and evaluated with UV light.

### Bisulfite sequencing

DNA was treated with bisulfite and purified for PCR as described previously [45]. The primers for *COX-2* sequencing were as follows: Forward primer 1, 5′-TTT GGA GAG GAA GTT AAG TG -3′ and reverse primer 1, 5′- CTC TCC CCT TAA AAA AAT TA-3′; Forward primer 2, 5′-AAG GGG AGA GGA GGG AAA AA-3′ and reverse primer 2, 5′- CGC CAA ATA CTC ACC TAT AT-3′. The PCR products were gel extracted with a Qiaquick gel extraction kit (Qiagen, Valencia, CA) and ligated into the pGEM-T easy vector by using the TA cloning system (Invitrogen, Carlsbad, CA). Ttransformed *Escherichia coli* DH5α cells were cultured overnight, and the plasmid DNA was isolated using a kit (Qiagen, Valencia, CA). At least 10 separate clones were chosen for sequence analysis.

### Chromatin immunoprecipitation (ChIP) assay

A chromatin immunoprecipitation (ChIP) assay was performed as described previously [46]. Briefly, 2 × 10^7^ cells treated with TPA were fixed with 1% formaldehyde at 37°C for 10 min and were then lysed on ice for 15 min. These lysed extracts were subjected to shearing by sonication. The soluble chromatin was subjected to immunoprecipitation with antibodies against different modified histones and other proteins as indicated. The primers for all ChIPs are available upon request.

### RNA interfence (RNAi)

RNA interference was performed as mentioned [47]. The sequences of RNA interference (RNAi) oligonucleotides for controls (non-specific) and c-Fos were as follows: non-specific siRNA, TTC TCC GAA CGT GTC ACG T; c-Fos siRNA, CAT CTG TGA ATG ATA ATT. These RNAi oligonucleotides were transfected into H719 and H460 cells by using the Lipofectamine 2000 transfection kit (Invitrogen, Carlsbad, CA) according to the manufacturer's instructions. Cells were harvested after 48 hours of transfection and subjected to Western blotting and a ChIP assay, respectively.

### Histone extraction, total protein extraction and western blotting

To identify histone modifications, acid extraction of histone was performed as previously reported [48]. To detect other proteins, cells were extracted with lysis buffer (50 mM Tris-HCl, 250 mM NaCl, 5 mM EDTA, 50 mM NaF, 1.5 mM PMSF and protease inhibitors cocktail). Equal amounts of protein were size fractionated on 6.0 to 15.0% SDS-PAGE gel. The antibodies used were anti-COX-2 (sc-19999, Santa Cruz), anti-c-Fos (ab7963, Abcam), anti-c-Jun (sc-44, Santa Cruz), anti-KMT3A (ab31358, Abcam), anti-KMT3B (17-10264, Merck Millipore), anti-KDM2A (ab31739, Abcam), anti-KDM2B (ab108276, Abcam), anti-KDM4A (ab70786, Abcam), anti-KDM4B (ab80473, Abcam), anti-NF-κB (sc-372G, Santa Cruz), anti-p300 (H-272, Santa Cruz), anti-CEBPβ (sc-150, Santa Cruz), anti-H3K4me1/2/3 (ab8895/ab7766/ab1012, Abcam), anti-H3K9me1/2/3 (ab9045/ab1220/ab8898, Abcam), anti-H3K27me1/2/3 (ab113671/ab24684/ab6002, Abcam), anti-H3K36me1/2/3 (ab9048/ab9049/ab9050, Abcam), anti-H3K79me1/2/3 (ab2886/ab3594/ab2621, Abcam), anti-H4K20me1/3 (ab9051/9053, Abcam), anti-histone H3 (ab131711, Abcam) and anti-β-actin (sc-1615, Santa Cruz).

### Co-immunoprecipitation (Co-IP)

H719 and H460 cells were treated with TPA for 2 hours at 100 ng/mL and then harvested and lysed in lysis buffer (1% NP-40, 150 mM NaCl, 50 mM Tris, 0.05% SDS, 1 mM PMSF, and a 1% cocktail of protease inhibitors) on ice for 20 min. Antibodies were then added to the supernatant on ice for 1 hour. The protein was analyzed by Western blotting with different antibodies. Antibodies used for Co-IP are available upon request.

### GST pull-down assay

The His fusion proteins were expressed in bacteria induced with isopropyl-β-D-thio-galactoside and purified. Equal amounts of His fusion proteins were incubated with glutathione-Sepharose 4B beads (GE Healthcare) and then washed three times with TEN buffer (20-mM Tris at pH 7.4, 0.1 mM EDTA, and 100 mM NaCl). GST-KDM2A purified from bacteria was incubated with the His fusion proteins. The beads were washed three times with TENT buffer (0.5% Nonidet P-40, 20 mM Tris at pH 7.4, 0.1 mM EDTA, and 300 mM NaCl) and analyzed by Western blotting with anti-GST antibody and Coomassie brilliant blue staining.

### FRET detection by fluorescence lifetime imaging microscopy (FLIM) analysis

For the FRET assay, HEK293T cells were cultured for 48 hrs after transfection with pECFP-c-Fos and/or pEYFP-KDM2A plasmids, on poly-D-lysine-coated coverslips (Fisher Scientific Microscope Cover Glass, 12-545-80, Hampton, NH, USA) and TPA was added for 2 hours. Cells were fixed by 4% paraformaldehyde and FLIM analysis was performed using a Leica TCS SP8 SMD scanning confocal microscope (Leica Microsystems, Wetzlar, Germany; APO 64 × / NA = 1.4, donor activation wavelength 458 nm). The FRET assay was performed with TCS SP8 confocal software (Leica, Germany).

### Data analysis

Statistical analysis was performed to assess the difference between two groups under multiple conditions by one-way analysis of variance (ANOVA) using PRISM statistical analysis software (GraphPad Software, Inc., San Diego, CA). The Student's test was performed to assess the difference between two groups in these studies and differences were considered significant at *p < 0.05, **p < 0.01 or ***p < 0.001.

## SUPPLEMENTARY FIGURES


