# Supplementary material for: The transcription factor c-Fos coordinates with histone lysine-specific demethylase 2A to activate the expression of cyclooxygenase-2
Source: Oncotarget. 2015 Sep 25;6(33):34704–17. doi: 10.18632/oncotarget.5474 (PMC4741484; doi:10.18632/oncotarget.5474)
Supplement: Supplementary file 1 [file oncotarget-06-34704-s001.pdf]

SUPPLEMENTARY FIGURES

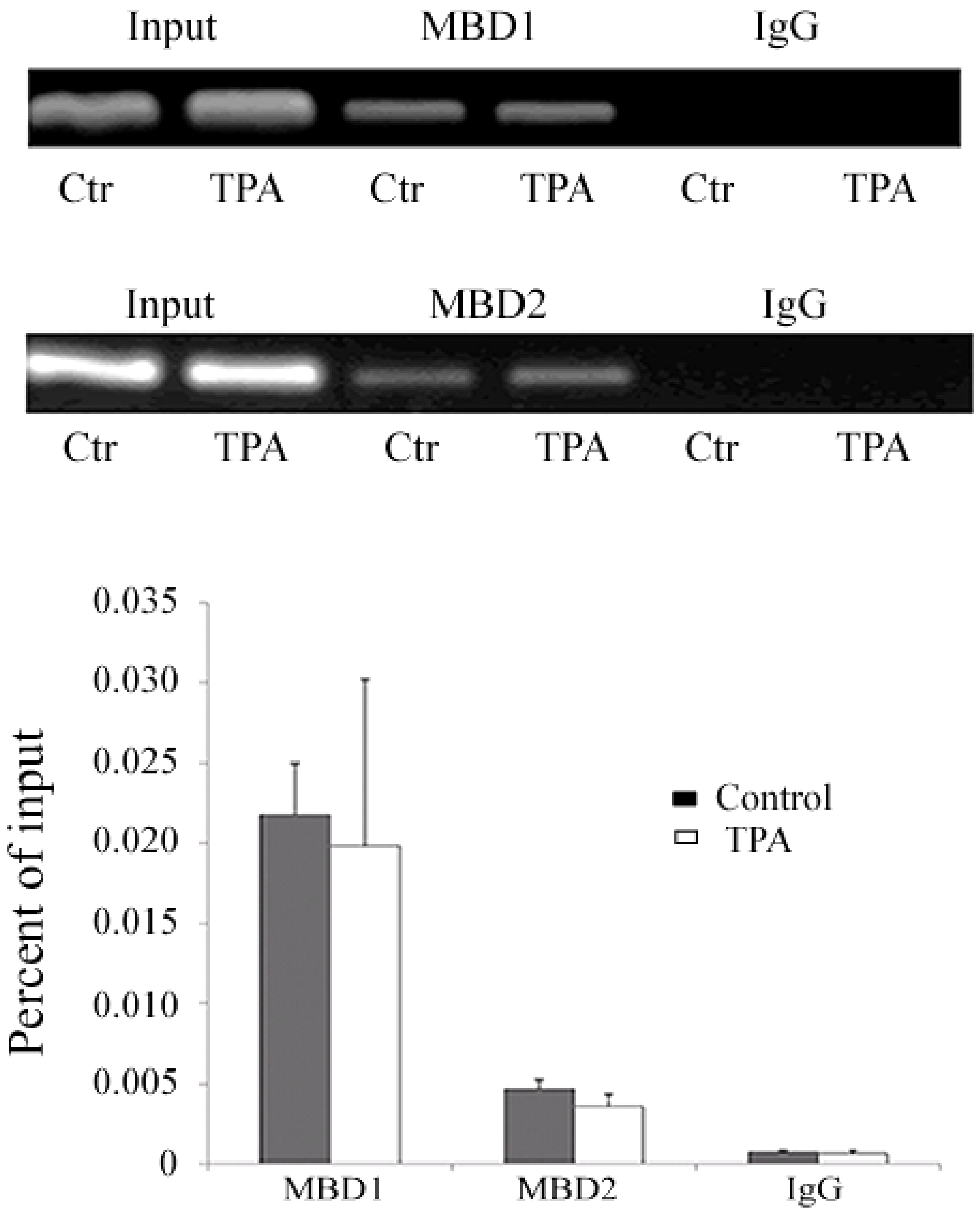

Supplementary Figure S1: The binding activity of methyl-CpG binding domain (MBD) proteins around the *COX-2* promoter. H719 cells were treated with 100 ng/mL TPA for 6 hrs and harvested for a ChIP assay to detect the binding activity of MBD1 and MBD2 around the *COX-2* promoter (the upper panel). The bands containing anti-IgG served as negative controls. ChIP-qPCR assay was also used to detect the changes in binding activity of MBD1 and MBD2 to the *COX-2* promoter after TPA treatment as outlined above (the lower panel).

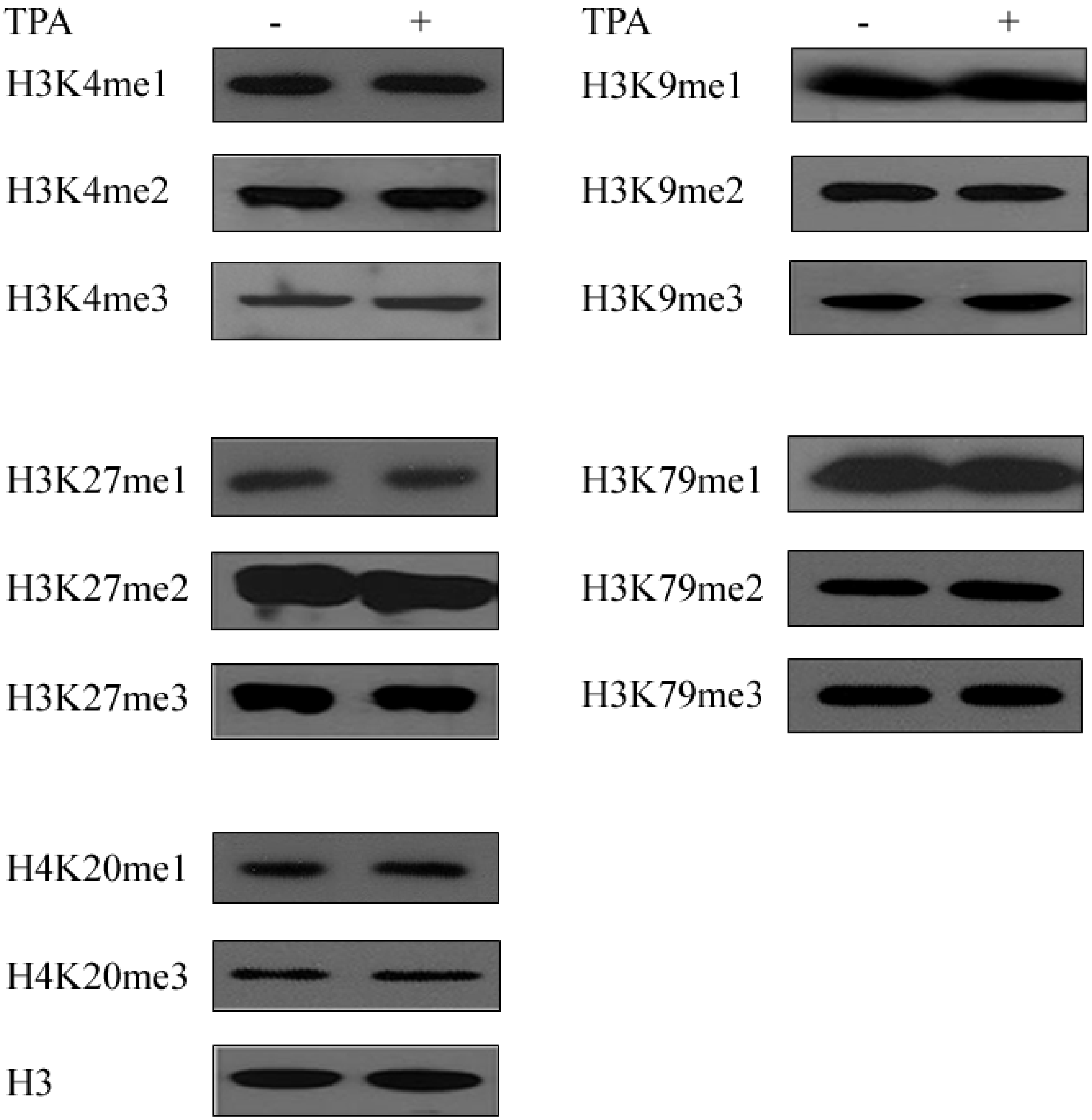

Supplementary Figure S2: The changes of the majority of histone methylation patterns after TPA treatment in H719 cells. H719 cells were treated with 100 ng/mL TPA for 6 hrs, and then the changes of different histone methylation patterns as shown in the figure were determined by Western blotting. H3 is shown as a loading control.

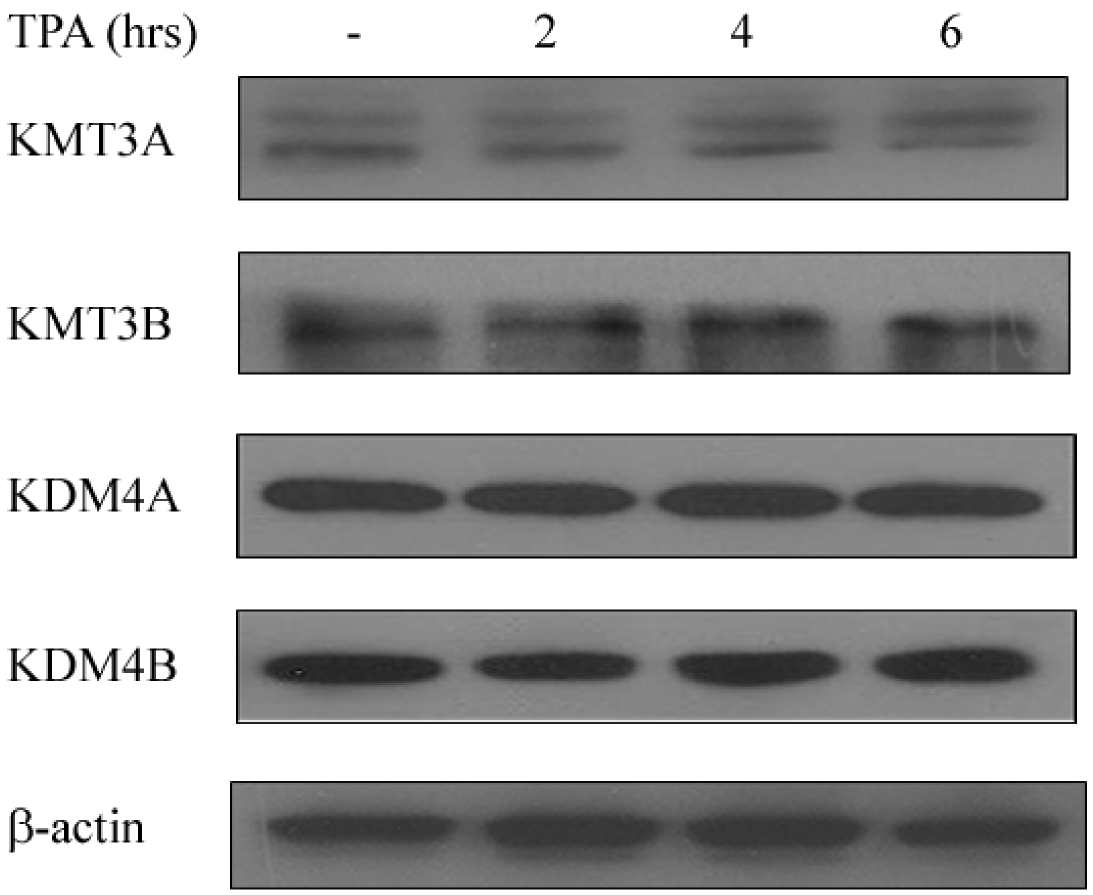

Supplementary Figure S3: The expression of methyltransferases and demethylases related to H3K36me2 after TPA treatment in H719 cells. H719 cells were treated with TPA at 100 ng/mL for various intervals (0, 2, 4 and 6 hrs), and the cells were then harvested for Western blotting to assess the expression of methyltransferases KMT3A, KMT3B or demethylases KDM4A and KDM4B, separately. β-actin was used as a loading control.

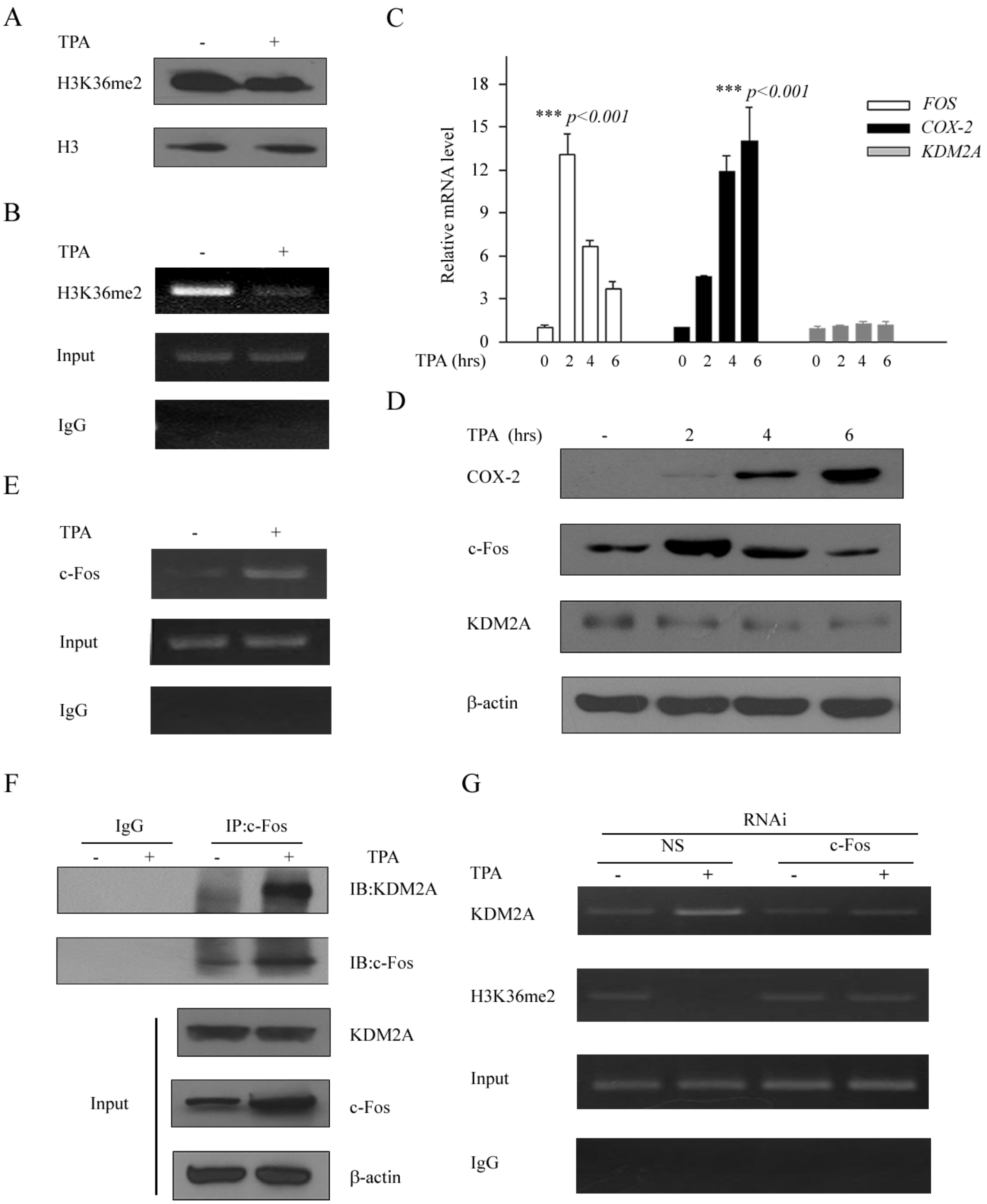

Supplementary Figure S4: The *COX-2* gene re-expression after TPA treatment in H460 cells. A. H460 cells were treated with 100 ng/mL TPA for 6 hrs, and the change in H3K36 me2 pattern was then determined by Western blotting. H3 is shown as a loading control. **B.** H460 cells were treated as outlined above, and harvested for a ChIP assay to detect the enrichment of H3K36 me2 around the *COX-2* promoter. The bands containing anti-IgG served as negative controls. **C.** H460 cells were treated with TPA at 100 ng/mL for various intervals (0, 2, 4 and 6 hrs), and the cells were then harvested for real-time PCR to detect the expression of *FOS*, *COX-2* and *KDM2A*. ***, $P < 0.001$. **D.** H460 cells were treated with TPA at 100 ng/mL for various intervals (0, 2, 4 and 6 hrs), and the cells were then harvested for Western blotting to assess the protein levels of COX-2, c-Fos and KDM2A. β-actin was used as a loading control. **E.** H460 cells were treated with TPA at 100 ng/mL for 2 hrs and harvested for a ChIP assay to detect the binding activity of c-Fos around the *COX-2* promoter. **F.** H460 cells were treated with TPA for 2 hrs at 100 ng/mL and the cells were then extracted for Co-IP using anti-c-Fos antibody, followed by Western immunoblotting with anti-KDM2A to assess the interaction between c-Fos and KDM2A. **G.** H460 cells were transfected with c-Fos siRNA or non-specific siRNA (NS) for 24 hrs and then treated with TPA for 4 hrs at 100 ng/mL. A ChIP assay was performed to detect the binding activity of KDM2A and the enrichment of H3K36me2 around the *COX-2* promoter.

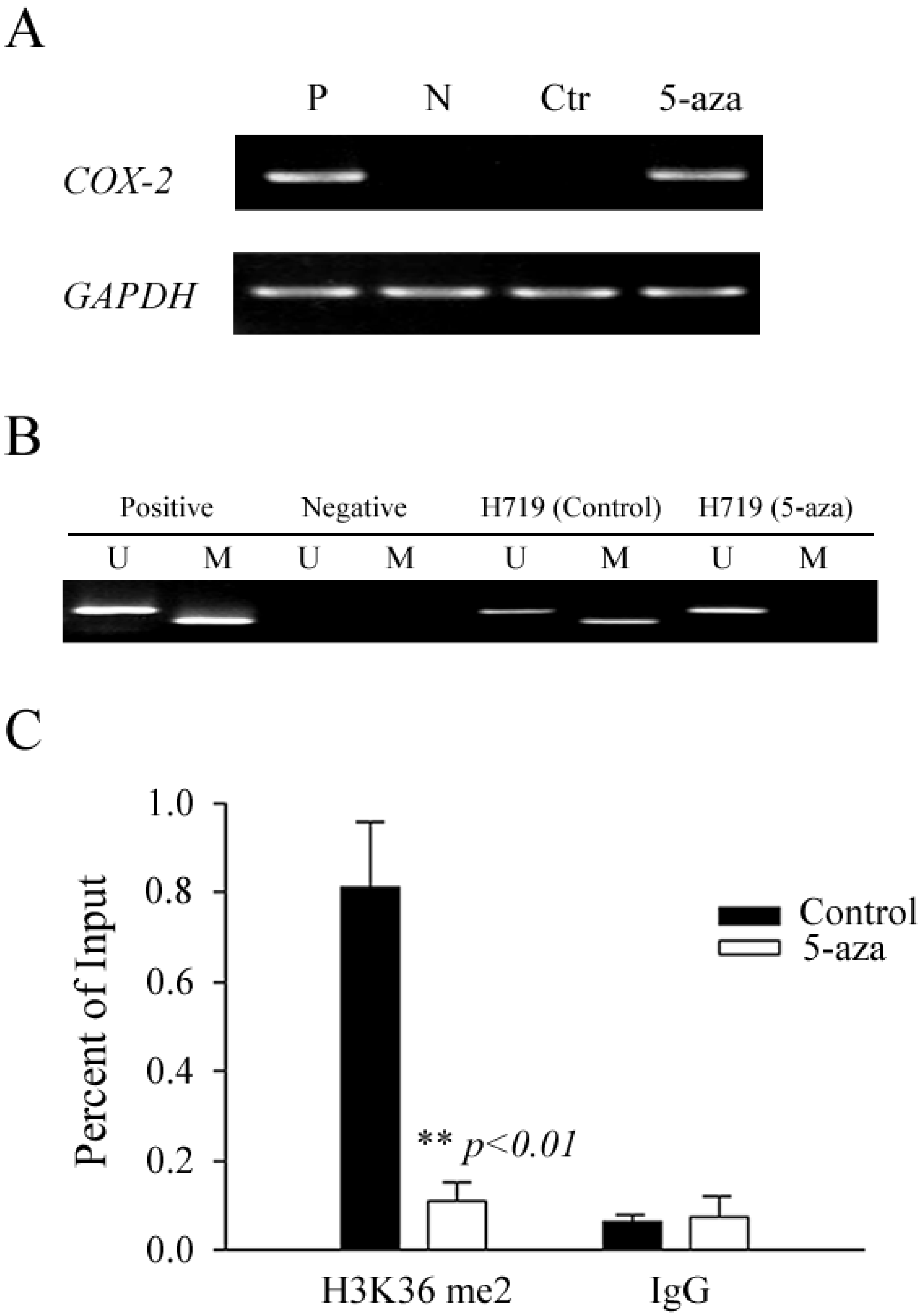

Supplementary Figure S5: The effect of 5-aza-2′-deoxycytidine on *COX-2* gene expression. A. H719 cells were treated with 5-aza-2′-deoxycytidine at 5μ (M for 96 hrs and RT-PCR was performed to detect *COX-2* mRNA levels after treatment. GAPDH was used as a loading control. P indicates a positive control, and N indicates a negative control. **B.** H719 cells were treated with 5-aza-2′-deoxycytidine as outlined above, MSP was used to detect the DNA methylation status of *COX-2* promoter before or after 5-aza-2′-deoxycytidine treatment in H719 cells. M indicates a methylated band and U indicates an unmethylated band. **C.** H719 cells were treated with 5-aza-2′-deoxycytidine as outlined above, a ChIP-qPCR assay was performed to detect the binding activity of H3K36me2 around the *COX-2* promoter. **, $p < 0.01$.

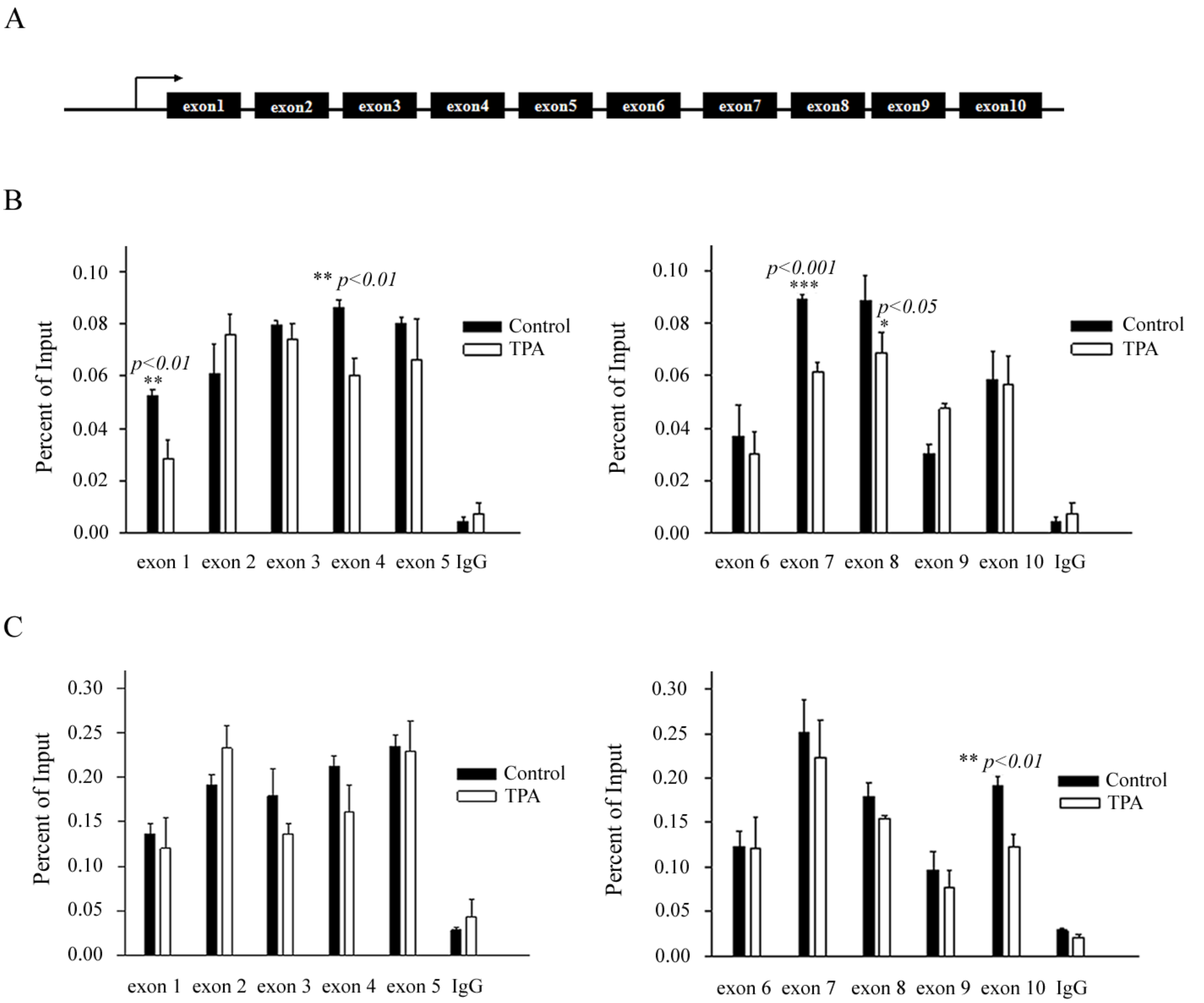

Supplementary Figure S6: The changes of H3K36 methylation in the transcribed region of *COX-2* gene. A. A schematic showing the transcribed region of *COX-2* gene. **B.** H719 cells were treated with TPA at 100 ng/mL for 6 hrs and then harvested for a ChIP-qPCR assay to detect the enrichment of H3K36 me2 around the transcribed region of *COX-2* promoter. *, $p < 0.05$; **, $p < 0.01$; ***, $p < 0.001$. **C.** H719 cells were treated with TPA at 100 ng/mL for 6 hrs and then harvested for a ChIP-qPCR assay to detect the enrichment of H3K36 me3 around the transcribed region of *COX-2* promoter. **, $p < 0.01$.

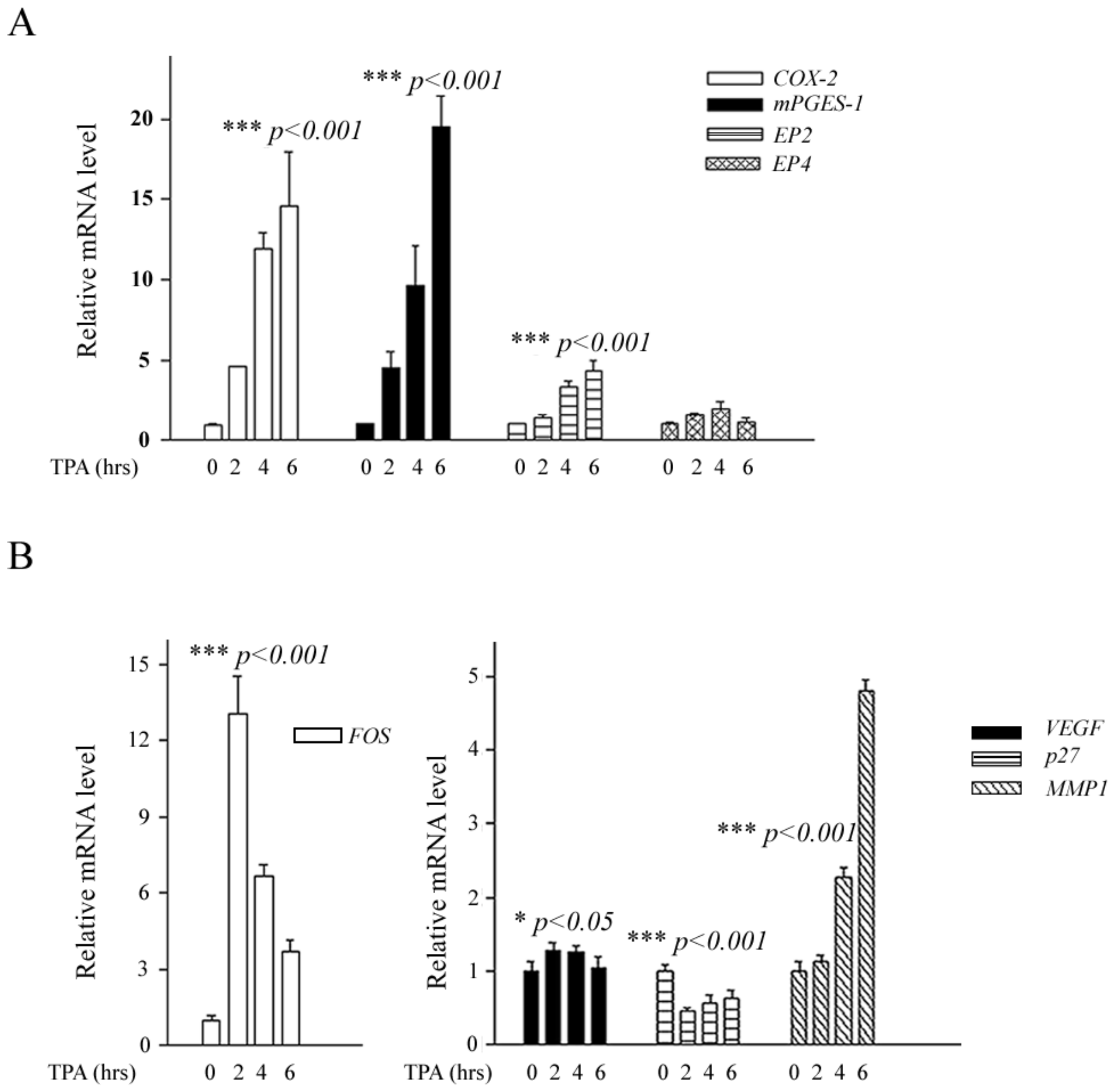

Supplementary Figure S7: The expression of down streams of COX-2 and c-FOS after TPA treatment in H460 cells. A. H460 cells were treated with TPA at 100 ng/mL for various intervals (0, 2, 4 and 6 hrs), and then harvested for real-time PCR to assess the expression of down streams of COX-2. **B, C.** H460 cells were treated as outlined above and then harvested for real-time PCR to detect the expression of several c-Fos targets. *, $p < 0.05$; **, $p < 0.01$; ***, $p < 0.001$.
